# Poly(piperazine-amide)/PES Composite Multi-Channel Capillary Membranes for Low-Pressure Nanofiltration

**DOI:** 10.3390/polym9120654

**Published:** 2017-11-28

**Authors:** Jan O. Back, Martin Spruck, Marc Koch, Lukas Mayr, Simon Penner, Marco Rupprich

**Affiliations:** 1 Management Center Innsbruck (MCI)—The Entrepreneurial School, Department of Environmental, Process & Energy Engineering, Maximilianstrasse 2, 6020 Innsbruck, Austria; jan.back@mci.edu (J.O.B.); martin.spruck@mci.edu (M.S.); marc.koch@mci.edu (M.K.); 2 Institute of Physical Chemistry, University of Innsbruck, Innrain 52c, 6020 Innsbruck, Austria; simon.penner@uibk.ac.at (L.M. & S.P.)

**Keywords:** multi-channel membrane, capillary membrane, interfacial polymerization, low-pressure nanofiltration, water softening

## Abstract

The mechanical stability of conventional single-channel capillary fibres can be improved in a multi-channel geometry, which has previously found application in ultrafiltration. In this work, multi-channel polyethersulfone (PES) capillary membranes comprising seven feed channels were successfully fabricated in an enhanced steam–dry–wet spinning process and coated on the inner surface with a thin polyamide (PA) layer via interfacial polymerization (IP). The coating procedure consisted of impregnating the support multi-channel capillary membranes (MCM) with an aqueous piperazine solution, flushing with nitrogen gas to remove excess droplets, and pumping an organic trimesoylchloride solution through the channels. Insights into the interfacial polymerization process were gained through the investigation of various parameters, including monomer ratio, contact time, and drying time. Membranes were characterised via scanning electron microscopy (SEM), atomic force microscopy (AFM), and filtration experiments. The optimisation of both the PES support membrane and IP process parameters allowed for the fabrication of composite MCM with an MgSO_4_ rejection of 91.4% and a solute flux of 68.8 L m^−2^ h^−1^ at an applied pressure of 3 bar. The fabricated composite MCM demonstrates that a favourable multi-channel arrangement can be upgraded with a PA layer for application in low-pressure nanofiltration.

## 1. Introduction

Due to its comparatively low operating pressures, high permeability and relatively low maintenance and operational costs, nanofiltration (NF) has evolved to a state-of-the-art technology in recent years [1]. The structure of NF membranes is less dense than that of reverse osmosis membranes, leading to molecular weight cut-off values in the range of 300–2000 Da and enhanced water permeate flux at the same operating pressure.

In terms of membrane geometry, tubular-shaped membranes such as hollow and capillary fibres provide benefits over the flat-sheet arrangement, as they show a high packing density and are self-supporting. Consequently, the application of external spacers is not necessary and the design of the final module is less complex. However, polymeric hollow and capillary fibres play a subordinate role in commercial NF so far, and to our knowledge only tubular NF membranes composed of ceramic materials have been proposed [2]. In order to increase the mechanical stability, particularly in the radial direction, with respect to single-channel fibres, a multi-channel configuration with a mean pore size down to 0.9 nm is available for ceramic NF membranes [3]. For ultrafiltration (UF), polymeric polyethersulfone (PES) membranes with seven feed channels are produced by inge GmbH (Multibore^®^, inge GmbH, Greifenberg, Germany) [4] with promising results of pilot tests published [5,6,7], and GE Water (ZeeWeed 700B, SUEZ, Trevose, PA, USA) [8]. More recently, Wan et al. [9] reported polyvinylidene fluoride (PVDF)-based seven-bore membranes with UF characteristics for water treatment.

Besides water applications, the concept of several channels in one fibre has been extended to other membrane processes. Wang and Chung [10,11,12] developed multi-bore PVDF hollow fibre with high permeate flux and superior mechanical stability for membrane distillation (MD). Lu et al. [13] also investigated membranes for MD, dip-coating tri-bore PVDF hollow fibres with Teflon^®^ (The Chemours Company, Wilmington, DE, USA) on the outer surface. Bettahalli et al. [14] presented tri-bore hollow fibres for air dehumidification. Hua et al. [15] reported thin-film composite (TFC) tri-bore hollow fibre membranes for pervaporation applications with enhanced separation performance and mechanical strength compared to conventional single-bore fibres. Forward osmosis (FO) characteristics were described by Luo et al. [16], who also tested composite tri-bore hollow fibres with a thin PA layer on the inner surface, whereas Li et al. [17] used tri-bore hollow fibres with a TFC layer on the outer surface for both FO and oil–water separation.

Polymeric NF membranes are generally composed of more than one material, of which the thin, active layer is fabricated via interfacial polymerization (IP). The TFC structure allows for independent control of both the support and active layer. As a result, TFC membranes can be optimised regarding permeate flux, retention and selectivity, while providing sufficient mechanical stability and compression resistance [18]. The support structure provides the required mechanical strength and exhibits low resistance to permeate flow. Most NF membranes with TFC design consist of an UF membrane as mechanical support, coated with an active polyamide (PA) layer. Due to economic aspects and its mechanical, thermal and chemical stability, PES or polysulfone (PSU) are often used as a support layer material [19].

IP with two reactive monomers is carried out by impregnating the microporous support membrane with an aqueous solution of a poly-functional amine, followed by adjoining an organic solution (often a hydrocarbon) of a poly-functional acid chloride. As the two solutions are non-soluble, and the final polymer is not soluble in either of the two phases, a thin PA film forms rapidly at the liquid–liquid interface, which coincides with the surface of microporous support [20]. Piperazine (PIP) and trimesoyl chloride (TMC) are commonly-used monomers for commercial NF membranes. In order to improve the performance of TFC membranes, several studies investigated different preparation conditions of composite flat-sheet and hollow fibre NF membranes [21,22,23,24,25], as well as the kinetics and mechanisms involved in the formation of the PA layer [26].

In general, the fabrication of composite hollow and capillary fibres seems more complex than flat-sheets when it comes to coating a uniform PA barrier on a substrate. The challenge of handling the aqueous and organic solutions and slow growth of the film are responsible for the increased complexity [27]. Regarding the surface to be coated, forming a consistent PA film on the outer surface of a tubular shaped membrane is much more sophisticated than coating the lumen surface. Despite the difficulties, a handful of studies [28,29] reported NF hollow fibre membranes with an active PA layer on the outer shell surface. Nonetheless, the IP process on the bore side of the hollow and capillary fibre membranes is thought to be better controllable in large-scale production, and several researchers reported dealing with PA film formation on the lumen surface of single channel fibres [30,31,32,33] as well as multi-channel fibres [15,16], all pointing out the importance of an optimised IP procedure.

In the first step of a typical IP procedure, regardless of the membrane geometry, the substrate is impregnated with an aqueous amine solution. Afterwards, excess solution and droplets have to be removed from the surface before exposure to the organic acid chloride solution, as otherwise the formed PA skin will not adhere sufficiently to the support membrane and defects are generated. For flat-sheet membranes, this issue is avoided by a rubber roller or an air knife, but these methods are rather impractical for tubular shaped membranes. Consequently, for tubular membranes the IP procedure has to be modified to produce a uniform and defect-free PA layer. Verissimo et al. [34] showed that the passage of an inert organic liquid between the two monomer solutions improved the formation of a PA active layer on the lumen surface of hollow fibres. They were able to reduce the formation of pin-holes in the film and improve the adhesion between the PA layer and the support material. A study by Korikov et al. [35] showed that the favourable combination of all factors controlling the IP process (i.e., monomer concentrations, reaction time, the removal of excess droplets, hydrophilization of the fibre and heat treatment) was important for the PA layer formation on polypropylene hollow fibres. Yang et al. [32] reported an adapted IP procedure for composite NF membrane preparation and removed excess PIP solution from the inner surface by flushing nitrogen gas through the lumen of the fibre. Their results showed that a defect-free PA skin was formed on the lumen surface by the modified process.

However, coating an intact PA layer onto the channel surfaces of a multi-channel capillary membrane (MCM) is more complicated due to the multiple channel arrangement. In a previous work [36], we applied a first generation of MCM with a relatively irregular shape and inferior mechanical stability to test the applicability of IP to the multi-channel configuration. The retention values of mono- and divalent salts indicated that IP had taken place to a limited extent, but also that a combination of inadequate support and a poor understanding of the IP process in the multi-channel arrangement led to defects in the formed PA layer. For successful application in NF, more reliable, defect-free support membranes and an elucidation of parameters controlling IP in MCM are required. Therefore, the first objective of this work was to apply principles from hollow-fibre production to PES MCM, in order to provide a defect-free substrate membrane with sufficient mechanical stability and a low membrane resistance to flow. Afterwards, various controlling parameters of the IP process, such as monomer concentration, reaction and drying time were investigated systematically, in order to further the understanding of IP in this particular membrane design and eventually to improve NF separation characteristics.

## 2. Materials and Methods

### 2.1. Chemicals and Instruments

As support structure, multi-channel membranes were prepared from a spinning dope containing PES (Veradel 3100P, Solvay Specialty Polymers, Düsseldorf, Germany), polyvinylpyrrolidone (PVP-K90) from Carl Roth (Karlsruhe, Germany), propane-1,2-diol (PD) from Sigma-Aldrich (Vienna, Austria), and *N*,*N*-dimethylacetamide (DMAc) from Sigma-Aldrich. Ethanol (≥96%) obtained from Carl-Roth and tap water were used as an internal coagulant.

A sodium hypochlorite solution (Carl Roth) with 12% free chlorine was diluted with deionised water and used as the post-treatment agent. PIP and TMC supplied from Sigma-Aldrich were used as reactive monomers for the interfacial polymerization process. Triethylamine (TEA) from Sigma-Aldrich was used as acid acceptor for the IP reaction. *n*-hexane from Merck Millipore (Vienna, Austria) was used as the solvent for the acid chloride.

MgSO_4_·7H_2_O obtained from Merck (Vienna, Austria) was used for the salt retention experiments. The salt concentration in the aqueous feed solution (deionised water) was adjusted to 3000 mg L^−1^ and the relative drop in the retention experiments was measured via electrical conductivity (WTW cond 315i, Zeller, Hohenems, Austria). Additionally, sucrose was used to evaluate the retention of the fabricated composite MCM. The concentration of sucrose was detected by a total organic carbon analyser (TOC-VCPN) from Shimadzu (Korneuburg, Austria). The feed temperature was kept at 25 ± 1 °C with a heating and cooling jacket.

The cross-sectional morphology of the prepared composite MCM was studied by SEM (JEOL, NeoScope JCM-5000, Freising, Germany). Fibres were cryofractured under liquid nitrogen to obtain a smooth observation surface. Atomic force microscopy (AFM) was performed using a Veeco Instruments Dimension 3100 system (Mannheim, Germany) with a Nanoscope IVa (Veeco Instruments) controller to study the surface structure of the fabricated MCM and composite MCM. The AFM was operated in tapping-mode at room temperature in air, and microfabricated probes of phosphorous-doped silicon with cantilever resonance frequencies around 300 kHz and a spring constant around 50 N/m were used.

### 2.2. Preparation of PES Multi-Channel Membranes

PES multi-channel capillary membranes were prepared in a steam–dry–wet spinning process. The detailed preparation parameters for MCM (Table 1) are the result of an internal optimisation procedure not expounded here. Major changes in the preparation routine since our last study [36] include variation of the polymer solution composition, implementation of a steam gap, variation of the bore fluid, design of a new spinning nozzle, and post-treatment of the fibres.

The polymer solution was transported to the spinneret by a pulse-free piston pump. The spinning dope was degassed before use to avoid structural defects caused by gas bubbles. For an assessment of the support membrane, mixtures of tap water and ethanol, as well as pure tap water, were used as an internal coagulation medium in the spinning process at a temperature of 30 °C. A continuous MCM preparation process was realised by a wind-up unit in the coagulation bath. The nascent fibres were not stretched in addition to gravity, i.e., the take-up speed was equal to the velocity of free-falling fibres. The total applied spinning gap length was 15 cm, comprising a steam cylinder with a length of 12 cm, and air gaps of 1 and 2 cm above and below the cylinder, respectively. The steam was generated electrically and supplied to the cylinder (4 cm diameter) from three sides so ensure a complete encasement of the nascent fibre.

**Figure 1:**
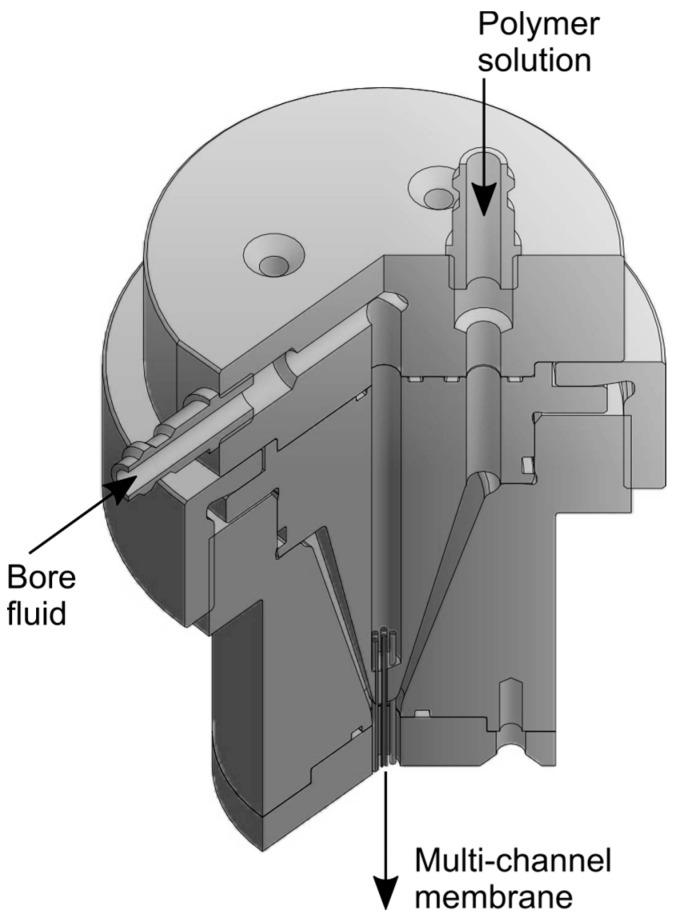
Sectional view of the multi-channel spinneret with seven bore fluid needles.

For the design of the spinning nozzle, it was assumed that axisymmetric dope flow in the spinning nozzle is advantageous due to control of elasticity-based instabilities and molecular orientation. As directional changes lead to uncontrollable shear stresses in the spinneret and affect the rheological characteristics of the polymer dope [37], deflection of the polymer solution inside the spinneret is kept at a minimum. Figure 1 shows a sectional image of the spinneret used for the fabrication of MCM. The dimensions of the spinneret were 4.8 mm for the diameter of the outer orifice and a diameter of 0.9 mm for each of the seven bore fluid needles. One needle was located at the centre, while six needles were placed concentrically at a radius of 1.5 mm. The needles had a wall thickness of 0.25 mm each. The stabilization distance for the polymer solution at the bottom of the nozzle was 10 mm.

The fibres were removed from the coagulation bath after five minutes and transferred to post-treatment tanks. The fabricated capillary membranes were stored in an aqueous hypochlorite solution (5000 ppm) for three days at room temperature in order to remove pore-building PVP from the membrane matrix and enhance permeability. Prior to further processing, the post-treated fibres were rinsed with water to remove residual bleach solution.

### 2.3. Preparation of Composite MCM

**Figure 2:**
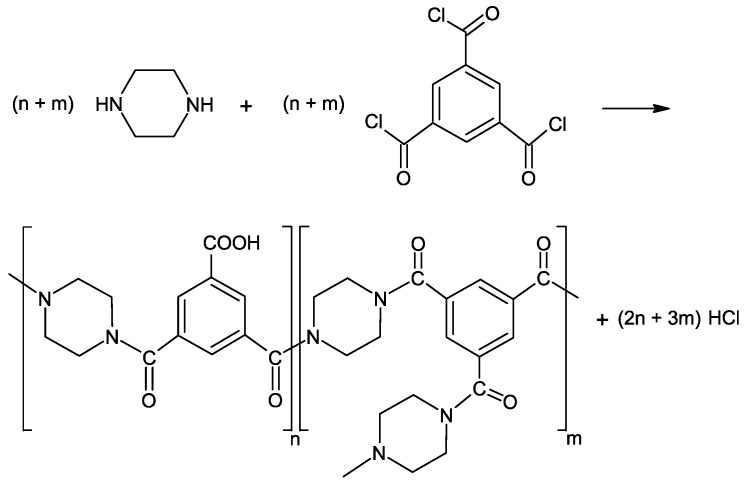
Reaction scheme of polyamide formation from piperazine (PIP) and trimesoylchloride (TMC) monomers; adapted from [38,39].

The active PA layer of the composite MCM was fabricated on the inner surfaces of the seven feed channels through IP. PIP and TMC were used as reactive monomers and TEA was used as the acid acceptor. Figure 2 shows the reaction scheme and the PA structure formed in the IP process of PIP and TMC.

**Figure 3:**
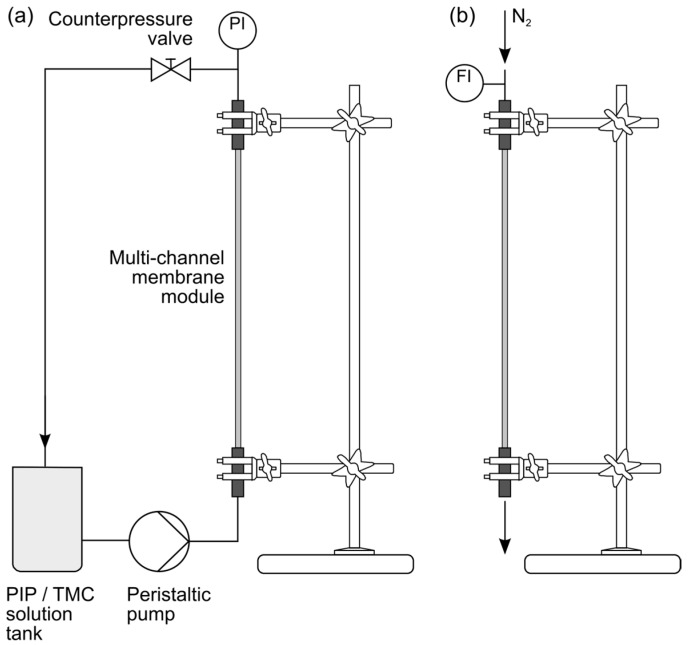
Preparation process of composite MCM; (**a**) processing of liquid PIP/TMC solutions; (**b**) flushing with nitrogen gas.

Wet MCM fibres were processed to modules containing a single multi-channel membrane with a length of approximately 54 cm, and fixed vertically before the coating procedure (see Figure 3). The aqueous solution containing defined mass fractions of PIP (0.2–0.8 wt %) and TEA was pumped bottom-up through the lumen of the MCM with a peristaltic pump at selected time periods. Subsequently, the excess amine solution was removed from the channel surface by flushing it top-down with nitrogen gas at a flow rate of 10 L min^−1^. The gas purging was a critical part of the modified IP process, as previously shown by Yang et al. [32]. In the case of the flow rate being too low, droplets of the aqueous amine solution could remain on the channel surface and lead to defects in the PA film, whereas a too strong gas purge could drain the surface and result in insufficient polymerization.

After N_2_ flushing, a TMC/*n*-hexane solution (0.075–0.6 wt %) was pumped through the lumen to induce the IP reaction. Before testing, the composite MCM fibres were dried in a vertical position at room temperature. The preparation procedure of composite MCM is summarized in Table 2.

## 3. Results and Discussion

### 3.1. Support Membrane

Support MCMs, prepared from PES in a steam–dry–wet spinning process, were subject to optimisation towards high flux and mechanical stability. The resulting preparation procedure is illustrated in Table 1. Particularly, the design of a new spinneret for MCM fabrication (Figure 1) has helped to produce suitable support membranes for IP due to axisymmetric dope flow and minimum deflection of the polymer solution [37].

To investigate the influence of bore fluid composition, fibres fabricated with pure water (subsequent identification (ID): BF W100) were coated in the described IP process and compared with coated fibres fabricated with 20 wt % ethanol/80 wt % water (ID: BF W80E20). Table 3 describes details of the IP process, as well as the results from flux and retention measurements. Both uncoated fibres show no salt rejection due to their UF characteristics. The higher permeate flux of the uncoated membrane prepared with pure water is attributed to fast demixing in the phase inversion process, whereas delayed demixing takes place when fractions of organic additives (ethanol) are added to the internal coagulation medium, resulting in denser support structures and lower flux [40]. The preparation procedure of support MCM was relatively robust, as independent preparation of MCM resulted in a relative standard deviation of permeate flux at 1 bar of 4.4% (*n* = 4, post-treatment (PT) in 5000 ppm NaClO: one day) for BF W100 and 3.9% (*n* = 3, PT one day) for BF W80E20, respectively. The salt rejection results of the coated fibres primarily indicate that IP was successful, underlining the potential of composite MCM for NF applications. Moreover, the surface of ethanol-based MCM seems more suitable for IP, pointing to denser and defect-free PA films on the inner channel surface compared to water-based MCM. Accordingly, ethanol-based support MCM (i.e., bore fluid composition 20 wt % ethanol/80 wt % water) have been used in further investigations. The IP procedure was also considered robust enough for further investigation, as preliminary results showed relative standard deviations of retention and flux of 0.5 and 0.2%, respectively (*n* = 2, 0.4 wt % PIP, PIP/TMC ratio = 6.2, PIP contact time = 100 s, TMC contact time = 30 s, drying time = 15 min, shown in Section 3.4), and 2.2 and 1.0%, respectively (*n* = 2, 0.4 wt % PIP, PIP/TMC ratio = 6.2, PIP contact time = 60 s, TMC contact time = 30 s, drying time = 15 min, not shown in figures below).

**Figure 4:**
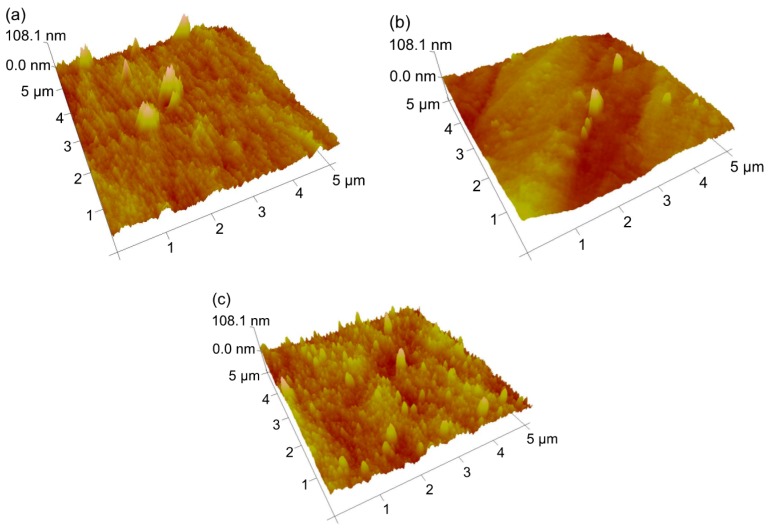
Three-dimensional atomic force microscopy (AFM) images of the inner surface of fabricated MCM; (**a**) uncoated MCM with ID W80E20; (**b**) uncoated MCM with ID W100; (**c**) composite MCM with ID NF2.

In an attempt to analyse PA layer formation and adherence in MCM, AFM measurements of the inner channel surface were carried out (Table 3 and Figure 4). These reveal similar root mean square roughness for the tested support membranes, and slightly higher roughness for IP-coated membranes. This is consistent with a study by Misdan et al. [41], who observed higher roughness of PA-coated membranes. Moreover, in a previous study [40] on the effect of coagulation medium composition, it was not possible to identify a clear trend in the surface structures with the addition of ethanol to the coagulation bath, which is confirmed in the presented AFM plots and data.

However, it is noted that cryofracture in the liquid nitrogen (preparation procedure for AFM and SEM) may have caused partial detachment of the PA layer, as pointed out in SEM discussions in Section 3.3. Hence, it cannot be ruled out that AFM plots and measurements represent the bare support membrane without the PA layer, even though salt rejection data clearly show that an intact PA film was formed in the IP process. This precludes further conclusions from the AFM results and calls for stronger PA adherence, e.g., via prior support surface functionalisation [42], to ensure the validity of the AFM data.

### 3.2. Drying Time of Composite MCM

**Figure 5:**
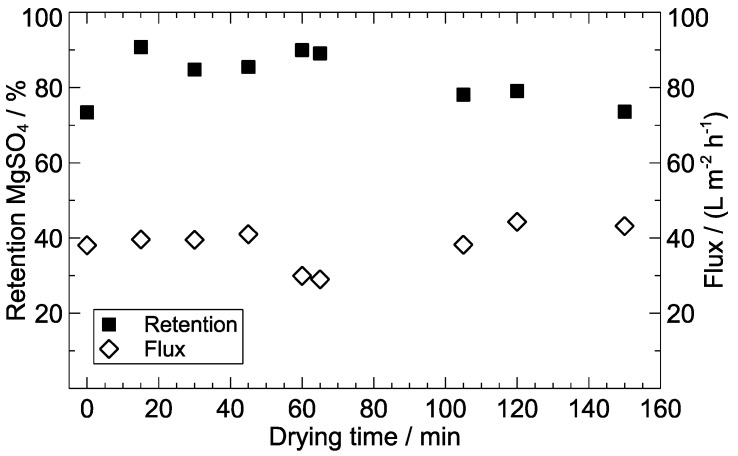
Effect of the drying time on the performance of composite MCM; aqueous amine solution: 0.4 wt % PIP, 0.15 wt % TEA; organic solution: 0.15 wt % TMC; ratio PIP/TMC = 12.5; feed solution: 3000 mg L^−1^ MgSO_4_; TMP = 2 bar.

As poly(piperazine-amide) growth occurs primarily in the organic phase [43], the freshly formed PA layer is located on a thin film of water after the reaction. Hence, water film removal may be required within the post-treatment procedure to guarantee sufficient linking with the PES support MCM [26]. Drying after IP and before characterization was carried out at room temperature with MCM fixed in a vertical position, whereas all other manufacturing parameters (concentrations, monomer ratio and reaction time) were kept constant. The flushing time for the aqueous and organic solution was 100 and 30 s, respectively, and ethanol-based support MCM were used. The values of MgSO_4_ retention and solute flux obtained at an applied transmembrane pressure of 2 bar are displayed in Figure 5 for drying periods between 0 and 150 min. Salt rejection was comparatively low when testing the composite MCM without prior drying. The highest salt rejection (90.8%) was obtained for a drying period of 15 min. Up to 65 min of drying, the retention decreased slightly. A stronger decline in rejection was found for extended post-treatment durations (<100 min). The solute flux was in the range of 29 to 44 L m^−2^ h^−1^ and was only slightly affected by the duration of drying.

The results indicate that a minimum drying period was required to stabilise the formed PA layer due to water film removal as well as the formation of an interpenetrating layer between the active layer and support membrane [26]. Also, densification of the dried PA layer and consequently, the shrinkage of membrane pores may take place [44]. On the other hand, rejection decreased when drying lasted too long. PA layers for NF swell up to 26.8% when exposed to water [45]. Hence, a possible explanation is excessive shrinkage of the dried PA layer, which led to the formation of defects in the active barrier or decreased the adherence of the film to the support membrane. Consequently, a drying duration of 15 min was selected for the further optimisation of other influencing parameters.

### 3.3. Contact Time of Monomer Solutions

In order to investigate the effect of monomer contact time inside the fibre, the duration of pumping the aqueous PIP and organic TMC solution through the ethanol-based support MCM was varied. A drying duration of 15 min at room temperature was used for all membranes as the post-treatment procedure. The values of salt retention and solution flux as a function of impregnation and reaction time are shown in Table 4.

Decreasing the impregnation time of the aqueous amine solution from 100 to 60 s had no significant impact on the MgSO_4_ rejection. This result is in accordance with a study by Yang et al. [32] on hollow fibre TFC membranes, showing that the surface of the support fibre was fully impregnated with the PIP solution after a short contact time. For different TMC contact times, the experimental data revealed no clear trend in salt retention. Paradoxically, flux increased with an extension of the TMC contact time. This result is in contrast with the work of Yang et al. [32], as they found a decline in water permeability for elongated reaction times and explained their observation with thicker PA layers. It may be concluded from the results of NF2 that the PA barrier was formed rapidly and a reaction time of 30 s was sufficient. Song et al. [26] showed that the formation of the PA layer occurred fast within the first 30 s of the polymerization reaction, followed by negligible changes with increased contact time. They argued that the dense PA barrier blocked the diffusion of the amine (*p*-phenylenediamine) and consequently, the reaction with TMC. On the other hand, the results by Yang et al. [32] indicated that the poly(piperazine-amide) layer was not dense enough to inhibit PIP diffusion. Consequently, the formation of the active skin layer was not affected by a self-limiting phenomenon and continued as the reaction time was extended. A possible explanation for the herein observed increase in flux with TMC contact time may be a partial, superficial stripping of the formed PA layer in the testing procedure, which in this case after 30 s seems to not grow any further.

**Figure 6:**
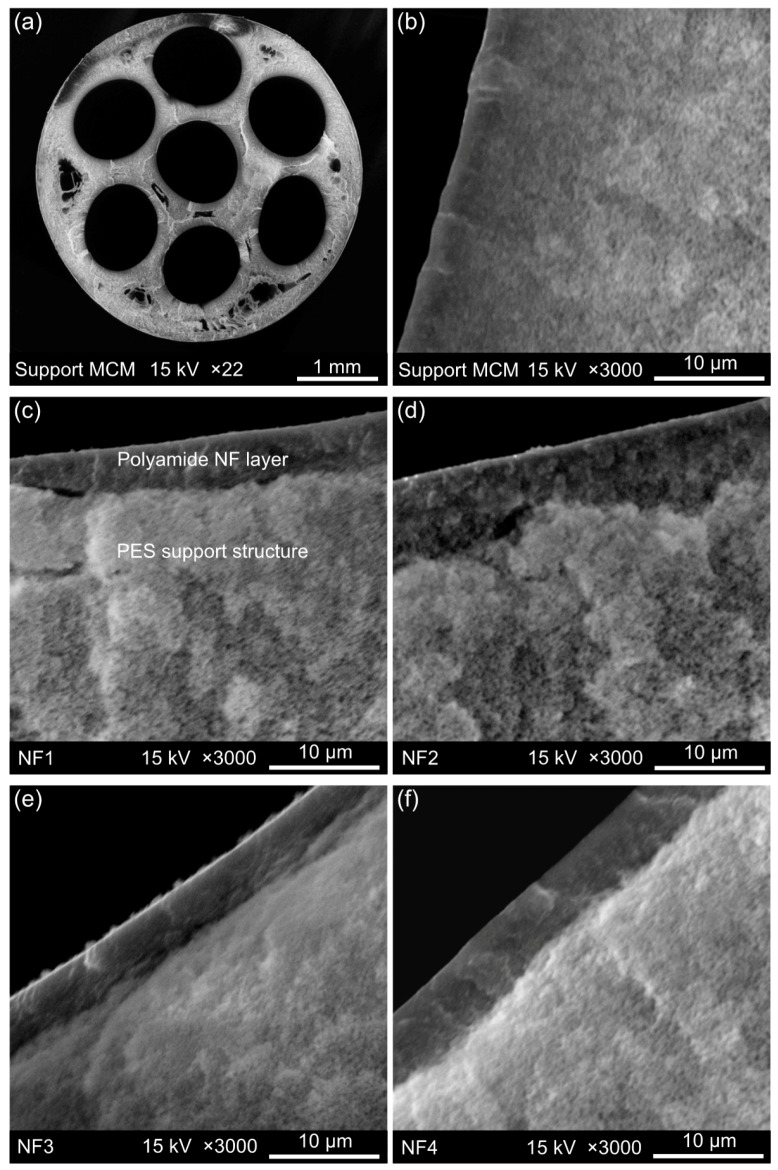
Cross sectional scanning electron microscope (SEM) images of (**a**) support MCM; (**b**) uncoated MCM as support material; (**c**) composite MCM NF1; (**d**) composite MCM NF2; (**e**) composite MCM NF3 and (**f**) composite MCM NF4.

The cross section of the uncoated support fibre and coated composite MCM was analysed by SEM. Images of the MCM fibre before and after PA layer formation on the channel surface are presented in Figure 6. The difference between Figure 6b and Figure 6c–f confirms that a dense layer was formed on the channel surface of the substrate MCM. A clear trend regarding the thickness of the PA film with changes in PIP/TMC contact time was not observed. It is acknowledged that the thickness of the coated PA barrier was not fully uniform and approximately in the range of ±1 µm for all tested composite MCM. Moreover, it was observed that cryofracture in liquid nitrogen (preparation procedure for SEM) may have caused partial detachment of the PA layer, revealing that active layer adherence to the PES support is rather weak and no covalent bonding between the layers exists, as alluded in previous AFM discussions in Section 3.1. The pore size on the lumen side was not detectable by SEM, either of support or coated membranes.

**Figure 7:**
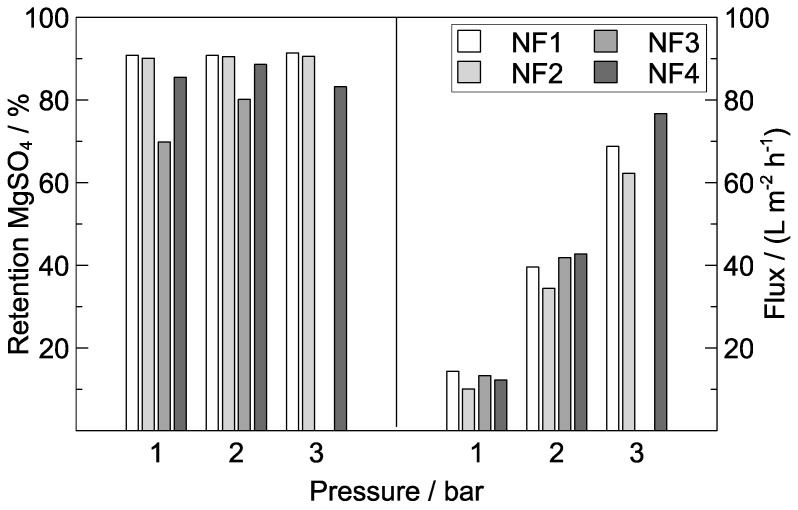
Permeate flux and MgSO_4_ (3000 mg L^−1^) retention of composite MCM over operation pressure; aqueous amine solution: 0.4 wt % PIP, 0.15 wt % TEA; organic solution: 0.15 wt % TMC; ratio PIP/TMC = 12.5.

Separation characteristics of the fabricated composite MCM with respect to operating pressure were investigated and the results are shown in Figure 7. It was observed that the permeate flux of membrane NF1 increased from 14.4 at 1 bar to 68.8 L m^−2^ h^−1^ at 3 bar, as rejection increased from 90.8 to 91.4%. MgSO_4_ rejection of membrane NF3 increased from 69.9 at 1 bar to 80.1% at 2 bar. Furthermore, rejection of sucrose (1000 mg L^−1^) was tested for NF3. Sucrose retention decreased from 87.1 to 79.8% as the operating pressure was raised from 1 to 2 bar (not shown in figure). The permeate flux obtained at an operating pressure of 3 bar exceeded 60 L m^−2^ h^−1^ for all composite MCM tested. These results demonstrate the NF characteristics of the tested composite MCM and reveal that the formation of a defect-free PA layer via IP was successful. Moreover, the applied pressure is relatively low compared to typical NF pressures (approx. 5–20 bar [46]), which qualifies composite MCM for low-pressure NF applications. In comparison to the NF membranes reported in the literature [21,30,32], composite MCM exhibits a higher permeability (22.9 L m^−2^ h^−1^ bar^−1^) with comparable salt retention (e.g., Fang et al. [30]: approx. 13 L m^−2^ h^−1^ bar^−1^ and 82% MgSO_4_ rejection at 2 bar and 1000 ppm feed solution). This is ascribed to the favourable design of the support MCM, which presents a low resistance to flow with still-high mechanical stability. Commercial PA TFC membrane manufacturers claim higher MgSO_4_ rejection, but at higher pressure and lower permeability: 8.9 L m^−2^ h^−1^ bar^−1^ (DOW FILMTEC (Edina, MN, USA), NF-90-400/34i, 2000 mg L^−1^ MgSO_4_, 4.8 bar, *R* = 97% [47]), 9.6 L m^−2^ h^−1^ bar^−1^ (SUEZ (Trevose, PA, USA), HL, MgSO_4_, 6.9 bar, *R* = 95% [48]), and 8.9 L m^−2^ h^−1^ bar^−1^ (Synder (Vacaville, CA, USA), NDX, 2000 mg L^−1^ MgSO_4_, 7.6 bar, *R* = 95% [49]), respectively.

### 3.4. Monomer Ratio

In order to gain more insights into the IP process in MCM, different ratios of PIP and TMC were used for the polymerization reaction. The ratio of the reactive monomers (here PIP/TMC) is a relevant factor for the preparation of composite membranes by IP. As Enkelmann and Wegner [50] pointed out, it affects the film thickness, and consequently, the performance of fabricated NF membranes. The molar ratio of PIP to TMC was calculated based on a solution volume of 50 mL used for each individual flushing procedure.

**Figure 8:**
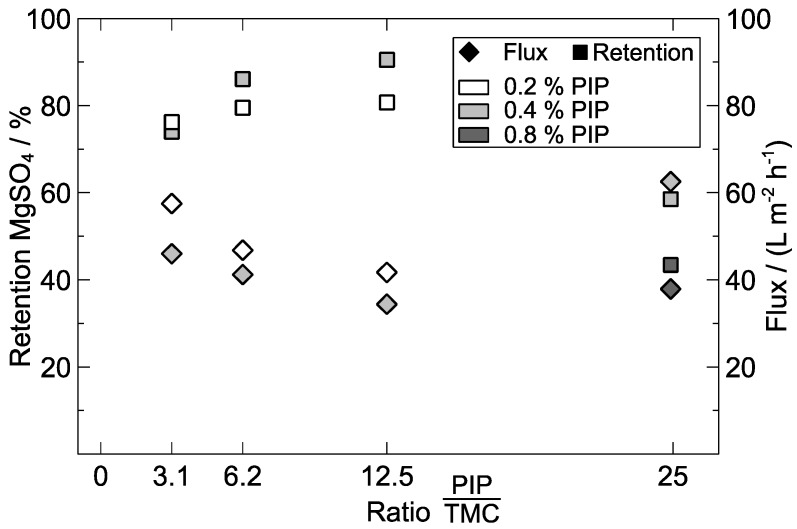
Effect of reactive monomer ratio (PIP/TMC) on the performance of composite MCM; concentration of TEA in the aqueous amine solution: 0.15 wt %; drying time: 15 min; feed solution: 3000 mg L^−1^ MgSO_4_; TMP = 2 bar.

The highest salt retention (90.5%) was found at a ratio of PIP to TMC of 12.5, as shown in Figure 8. Contact times for all composite MCM were 60 s for the PIP and 30 s for the TMC solution, and support membranes were ethanol-based. Mass fractions of 0.2 and 0.4 wt % PIP in the aqueous solution seemed particularly suitable in terms of salt retention and flux. At a mass fraction of 0.2 wt % PIP, a higher concentration of TMC in the organic crosslinking solution (lower ratio) led to an increase in flux and, in turn, to a decrease in salt retention, similar trends being observed for 0.4 wt % PIP. For the latter concentration, only at a ratio of 25 membrane did permeability start to increase, and salt retention decreased sharply. Hence, the flux of composite multi-channel fibres soaked with 0.4 wt % PIP may be described by a U-shaped behaviour at monomer ratios of 3.1–25, whereas an inverse trend was observed for MgSO_4_ retention. These results are in accordance with a study from Zhang et al. [21], who concluded from filtration experiments that there is an ideal PIP/TMC ratio at the peak of an inverted U. Liubimova et al. [51] observed two ideal monomer ratios deviating from 1:1 in a different PA system and attributed them to predominantly linear or weakly crosslinked layers, which achieved higher selectivity than strongly-crosslinked PA. They also stated that a large excess of one of the monomers caused a strong decrease in functionality. Accordingly, the best rejection rate (90.5%) was obtained using 0.4 wt % piperazine in the aqueous solution and a monomer ratio of 12.5, possibly forming primarily linear poly(piperazine-amide) chains. The decrease in large PIP excess may be interpreted at the molecular level: a too high PIP concentration may cause a reactive group blocking of diffusing TMC molecules and consequently, a truncation of PA chain growth.

## 4. Conclusions

Composite MCM comprising comprised seven feed channels coated with an active PA barrier layer was successfully fabricated for NF in a modified IP process. Systematic parameter investigation enabled us to gain insights into the coating process of MCM. In a first step, PES/PVP support membranes were produced and optimised in a steam–dry–wet spinning process with a novel spinneret, showing that axisymmetric flow and minimum deflection of the polymer solution are paramount for successful MCM production. Furthermore, bore fluid composition investigations revealed that delayed demixing in the phase inversion process and consequently, denser support structures, enhance the suitability for coating via IP. Subsequent IP parameter analysis included studying drying time within the post-treatment, contact time of reactive monomer solutions, their concentrations and molar ratio.

The results demonstrate the influence of the drying period on NF performance, which was applied to eliminate the water layer underneath the PA film and stabilise the NF layer without causing PA shrinkage and delamination. A short impregnation time of the aqueous PIP solution (60 s) was sufficient, and subsequent PA film growth at the interface with the organic TMC solution occurred to a sufficient amount after 30 s. The identified optimum of PIP/TMC ratio (12.5) indicated predominantly linear PA chains under ideal conditions and monomer-scavenging effects at large PIP monomer excesses. The optimised composite MCM showed an MgSO_4_ retention of 91.4% and a solute flux of 68.8 L m^−2^ h^−1^ tested at 3 bar operating pressure. Morphological investigations via SEM as well as the higher surface roughness of coated MCM in AFM illustrated the formation of a dense PA layer on the inner surface of the support fibre, whereas partial detachment of the PA layer in the preparation procedure for SEM and AFM (cryofracture in nitrogen) showed the need for improved linking of the two layers, especially to ensure the stability of the PA layer in long-term operation. Overall, the superior multi-channel arrangement of polymeric membranes with enhanced mechanical stability may find new applications in low-pressure NF.

## Figures and Tables

**Table 1:** Dope composition, spinning parameters and post-treatment of produced MCM.

Parameter	Specification
Dope composition/wt %	PES/PD/PVP-K90/DMAc 17/15/5/63
Dope flow rate/(cm^3^ min^−1^)	68
Dope temperature/°C	Room temperature
Bore fluid composition/wt %	Water/ethanol (80/20); pure water
Bore fluid flow rate/(L h^−1^)	4.0
Bore fluid temperature/°C	30 ± 1
Coagulation bath	Tap water
Coagulation bath temperature/°C	30 ± 1
Total spinning gap/cm	15
Steam gap/cm	12
Fibre post-treatment	5000 ppm NaClO, three days

**Table 2:** Preparation procedure of composite multi-channel capillary membranes (MCM).

Step	Operation
1	Flushing with aqueous PIP/triethylamine (TEA) solution
2	N_2_ gas flushing for 6 min
3	Flushing with TMC in *n*-hexane solution
4	Post-treatment (drying)

**Table 3:** Effect of different bore fluid compositions, showing monomer ratio (PIP/TMC), reaction time (*t*), retention (*R*), and flux of uncoated and composite MCM; TMP = 2 bar.

ID	PIP	TMC	Ratio	*t*_(PIP)_	*t*_(TMC)_	*R*_(MgSO4)_	Flux	*R*_q_
	wt %	wt %		s	s	%	L m^−2^ h^−1^	nm
BF W100	-	-	-	-	-	0.0	617.1	13.7
NF 5	0.4	0.15	12.5	60	30	74.3	44.0	-
BF W80E20	-	-	-	-	-	0.0	479.3	13.0
NF2	0.4	0.15	12.5	60	30	90.5	34.4	15.5

**Table 4:** Effect of monomer reaction time (*t*) on the retention (*R*) and solute flux of composite MCM; aqueous amine solution: 0.4 wt % PIP, 0.15 wt % TEA; organic solution: 0.15 wt % TMC; ratio PIP/TMC = 12.5; feed solution: 3000 mg L^−1^ MgSO_4_; TMP = 2 bar.

No.	*t*_(PIP)_	*t*_(TMC)_	*R*_(MgSO4)_	Flux
	s	s	%	(L m^−2^ h^−1^)
NF1	100	30	90.8	39.6
NF2	60	30	90.5	34.4
NF3	60	45	80.1	41.9
NF4	60	60	88.6	42.8
